# Predicting Properties of Key Ingredients of Battery Solvents with Density Functional Theory and Adaptive Force Matching

**DOI:** 10.1021/acs.jctc.6c00942

**Published:** 2026-08-04

**Authors:** Yang Wei, Raymond Weldon, Feng Wang

**Affiliations:** Department of Chemistry and Biochemistry, 3341 University of Arkansas, Fayetteville, Arkansas 72701, United States

## Abstract

The performance of batteries is heavily influenced by the properties of their solvents, which play a crucial role in ion solvation, conductivity, and electrochemical stability. However, traditional force field models often fall short in accurately predicting these properties. This study investigates the potential of adaptive force matching (AFM) to predict a range of solvent-related properties using only electronic structure theory. To demonstrate this approach, we developed AFM models based on B3LYP-D3­(BJ) for three common molecules present in battery solvents: dimethylamine (DMA), dimethyl carbonate (DMC), and tetrahydrofuran (THF). Our results reveal that AFM models significantly outperform traditional force fields, including OPLS-AA (CM1A), GAFF2, and GROMOS 54A7, in predicting key properties such as density, heat of vaporization, viscosity, diffusion constant, boiling temperature, and free energy of vaporization. Notably, the average percentage error of AFM models is approximately 13%, substantially lower than that of traditional force fields (21% for GAFF2, the best-performing empirical model). These findings underscore the promise of AFM in predicting physical properties of battery solvents, with far-reaching implications for chemistry, materials science, and related fields where accurate predictions are essential for understanding complex phenomena and designing innovative materials and systems.

## Graphical abstract

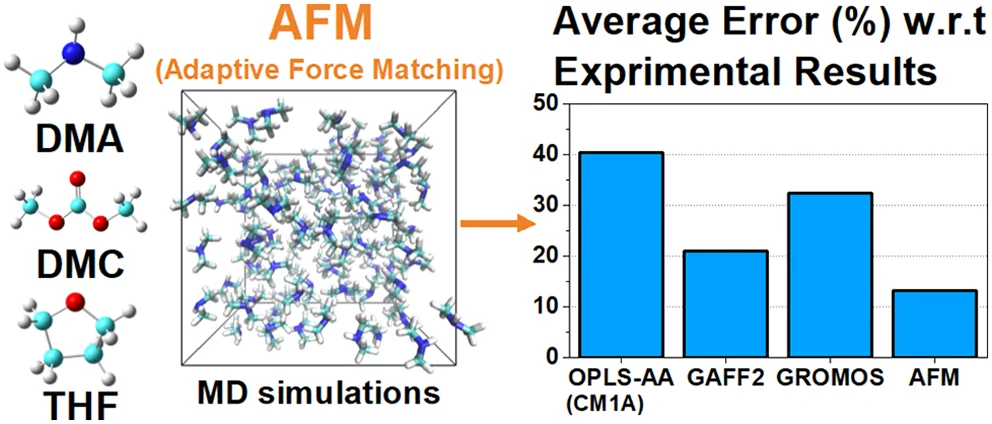

## 1 Introduction

The performance of a battery is profoundly impacted by the properties of its solvent, which plays a vital role in enabling efficient ion solvation, facilitating high molar conductivity, and maintaining electrochemical stability.^1–5^ Furthermore, an ideal solvent should possess a combination of desirable characteristics, including low melting temperatures and minimal vapor pressures, to ensure optimal battery operation across a broad temperature range.^1,3,6^ To rationally optimize battery chemistry, it is crucial to develop a detailed atomistic understanding of the complex interactions between electrolytes and solvents under operating conditions. Molecular dynamics (MD) simulations offer a powerful means of achieving this goal, as they can provide valuable insights into the behavior of these systems at the molecular level, which is challenging to obtain through current experimental techniques.

Numerous research groups have employed atomistic-scale simulations to investigate ion solvation and transport in common electrolyte solvents.^7–13^ However, the accuracy of these predictions is heavily dependent on the interaction potential utilized. Many existing models exhibit deficiencies; for example, the General Amber Force Field (GAFF)^14^ provides a good description of density but fails to predict viscosity with sufficient accuracy.^15^ This limitation is not unexpected, as most model development relies on fitting to experimental data to some extent. While empirical adjustments can improve agreement with target properties, such agreement may have benefited from fortuitous error cancellations rather than through a true representation of the underlying intermolecular interactions. Consequently, such models may be less reliable when predicting properties that were not explicitly fit. A more robust approach would be to develop simulations based solely on accurate first-principles potential energy surfaces. If these models can achieve sufficient accuracy for property prediction, they reflect a true prediction of the underlying electronic structure method. Such models will provide a more comprehensive and reliable understanding of battery chemistry, enabling more accurate predictions and improved design of battery materials.

The Car–Parrinello and Born–Oppenheimer molecular dynamics (CPMD and BOMD) methods are well-established techniques for electronic structure-based simulations,^16–18^ which have been successfully applied to study ion solvation and battery-related chemistry. However, these approaches are computationally intensive, as they require electronic structure calculations to be performed on the entire simulation box at each time step of the MD simulation. This limitation restricts the accessible time scales and system sizes that can be studied, making it challenging to explore complex phenomena that occur over longer periods or in larger systems. Furthermore, the high computational cost of these methods also limits the use of hybrid functionals, which offer improved accuracy but are more computationally demanding. Pure density-based exchange-correlation functionals often suffer more from charge delocalization errors, rendering them less robust for modeling polar solvents.

A promising alternative to the CPMD approach is adaptive force matching (AFM), which offers a powerful tool for developing molecular mechanics-based force field models.^19,20^ The AFM method involves fitting quantum mechanical (QM) forces, obtained from modeling QM clusters in a condensed-phase environment, to develop a force field model that iteratively refines toward QM accuracy. Notably, the environment for the QM clusters is modeled using the AFM potential being developed, allowing for a self-consistent and accurate representation of the system.

By leveraging QM clusters, AFM enables the use of more computationally intensive QM methods, such as second-order Møller–Plesset perturbation theory (MP2),^21,22^ which is essential for accurately predicting hydration free energies of ions^23–25^ and modeling weakly interacting systems, including CO_2_ and alkanes.^26–30^ A typical AFM model can be trained with only 1000 QM calculations; the resulting model can be used to simulate significantly larger systems over extended time scales, offering a substantial improvement in computational efficiency. Furthermore, AFM models can perform alchemical free energy calculations, providing a straightforward means of determining thermodynamic properties such as vapor pressures and boiling temperatures, which are crucial for understanding the behavior of complex systems.

In this study, we expand on the applicability of AFM by investigating its ability to accurately model common ingredients of battery solvents, including dimethylamine (DMA), tetrahydrofuran (THF) and dimethyl carbonate (DMC).^31–35^ These solvents exhibit intermediate polarity, bridging the gap between highly polar water^36^ and nonpolar alkanes,^30^ both of which have been previously studied using AFM. The chemical diversity of these molecules, spanning amines, ethers, and carbonates with varying contributions from dispersion and dipolar interactions, provides a challenging yet informative test case for the AFM methodology. By demonstrating the ability to accurately predict a range of experimental properties solely from electronic structure information, we further validate the efficacy of AFM in materials science applications.

Unlike traditional force fields, AFM models do not rely on combining rules or empirical fitting to experimental properties. Despite these philosophical differences, we compare the performance of AFM models to several well-established force fields, including OPLS-AA (CM1A),^37^ GAFF2,^38^ and GROMOS 54A7.^39^ Our results show that AFM models based on B3LYP-D3­(BJ) consistently outperform these traditional force field models in reproducing a range of properties, such as density, heat of vaporization, self-diffusion constant, viscosity, vaporization free energy, and boiling temperature.

The remainder of this paper is organized into five sections. Following the introduction, Section 2 provides a detailed description of the application of AFM to develop models for DMA, DMC, and THF. The methodology used to calculate various properties is outlined in Section 3. In Section 4, we present the results obtained using the AFM models and compare them to those from other force field models. Finally, a summary of the key findings and conclusions is presented in Section 5.

## 2 AFM Development of Models for DMA, DMC and THF

The AFM models are trained against density functional theory (DFT) calculations using the Becke-3-parameter Lee–Yang–Parr (B3LYP)^40–42^ exchange-correlation functional with the def2-TZVP basis set.^43^ To account for dispersion interactions, we utilize Grimme’s D3 approach with the Becke–Johnson (BJ) damping scheme.^44,45^ We picked B3LYP-D3­(BJ) due to its good performance in predicting hydration free energies of small molecules,^46^ although more advanced functionals^47–49^ or post-HF methods^26,50^ can be also be used with AFM. All three models will follow the same procedure.

In the AFM framework, each symmetry-unique atom in the target molecules is assigned a distinct atom type. The atom types assigned to the DMA, DMC, and THF molecules are illustrated in Figure 1. To initiate the AFM process, we select the OPLS-AA­(CM1A) force field as our initial guess, which combines the Lennard-Jones parameters from the OPLS-AA force field^51^ with improved charges generated by the LigParGen server using the 1.14CM1A-LBCC method.^52^ The initial simulation boxes for each target molecule are created using Packmol.^53^

**Figure 1:**
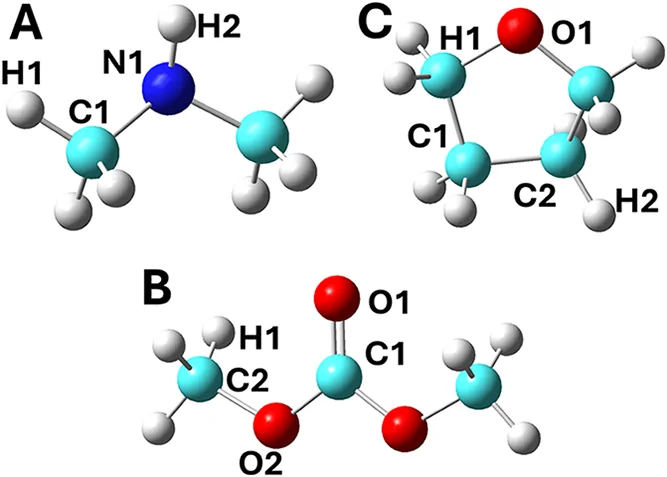
Atom types of (A). dimethylamine (DMA) (B). dimethyl carbonate (DMC) and (C). tetrahydrofuran (THF).

With AFM, dispersion parameters are typically fitted independently before the iterative procedure to determine other parameters. Since the QM method chosen is DFT, the dispersion will be fitted against D3­(BJ) dispersion. This fitting is performed using dimers of the target molecules. To ensure that these dimers are representative of dimer conformations in the liquid phase, we performed MD simulations in the liquid phase using the initial guess force field. A simulation box containing 1000 molecules was used to provide a sufficient size to allow for the extraction of dimers with large separations. After a brief equilibration period, the dimers were extracted from an NPT simulation at 1 bar and 280 K for DMA (its normal boiling temperature) and 298 K for DMC and THF.

For each target molecule, 840 snapshots were taken at intervals of 250 fs from the final 120 ps NPT simulation. Two sets of dimer conformations were extracted from these 840 snapshots: a uniform set and a random set. The uniform set consisted of an equal number of dimers extracted from each of the 0.1 Å wide windows between 5 and 12 Å. The random set comprised one dimer randomly selected from each of the 840 snapshots, also with a distance between 5 and 12 Å. The combination of these two sets resulted in a total of 1680 dimers for each target molecule, which were used for fitting the dispersion parameters.

In parametrization, the dispersion will be modeled with the short-range damped form introduced by Zheng and Wang^30,46^

1 $$V_{\mathrm{disp}}\left(r_{\mathrm{ij}}\right)=\frac{C_{6}}{r_{0}^{6}+r_{\mathrm{ij}}^{6}}+\frac{C_{8}}{r_{0}^{8}+r_{\mathrm{ij}}^{8}}$$

where *r*_0_ is the 0.6 times the sum of the atomic van der Waals radii, taken form the work of Tkatchenko,^54^ *r_ij_* is the distance between atoms *i* and *j*, and *C*_6_ and *C*_8_ are dispersion coefficients corresponding to the leading and higher-order terms of the London dispersion interaction, respectively. Dispersion interactions are only included between pairs of heavy atoms.

The short-range dispersion should always be attractive. If either *C*_6_ or *C*_8_ is fitted as a positive value, the higher-order *C*_8_ term will be eliminated, and the remaining dispersion terms will be refitted. If the remaining *C*_6_ term is still fitted to be positive, then both *C*_6_ and *C*_8_ terms will be eliminated to ensure that no positive *C*_6_ or *C*_8_ parameters remain in the final model.

After dispersion parameters are determined, AFM iterations are performed. Each iteration of AFM consists of three steps: the sampling step, the Quantum Mechanics/Molecular Mechanics (QM/MM) step, and the force-matching (FM) step. The first iteration of AFM is performed using the initial guess force field, which serves as both the basis for the sampling step and the Molecular Mechanics (MM) model in the QM/MM step. In subsequent iterations, the force field fitted in the previous iteration is used.

The sampling step is performed using MD simulations, which are conducted in a cubic box that is approximately 2.3 nm in each dimension. The number of molecules varies from 90 to 114, depending on the volume occupied by each molecule. The simulations are performed at two temperatures, with the higher temperature designed to better sample the repulsive wall of the potential. For DMA, the two temperatures are 280 and 313 K, while for DMC and THF, the temperatures are 298 and 338 K. Following a brief equilibration period, production trajectories are simulated for 1 ns under NPT conditions with a time step of 0.5 fs. The temperature is controlled using a Nose-Hoover thermostat^55^ with a relaxation time of 0.5 ps, and the pressure is controlled using an isotropic Parrinello–Rahman barostat^56^ with a relaxation time of 2.0 ps.

For the 313 K sampling simulation of DMA, an NVT simulation is performed instead, using the average volume from the 280 K NPT simulation. This is because 313 K exceeds the boiling temperature of DMA. Although an NPT simulation at this temperature might not necessarily induce vaporization of the liquid, we employ an NVT simulation as a precautionary measure to ensure stability.

For each temperature, 100 conformations are extracted from the final 500 ps of the trajectory, with a spacing of 5 ps, to be used as the training set for the QM/MM step. This results in a total of 200 conformations for each target molecule per generation of AFM.

The QM/MM step employs the B3LYP-D3­(BJ) method with Coulombic embedding for the treatment of MM particles. The def2-TZVP basis set is used for these calculations. A typical implementation of AFM involves dividing the QM region into two zones: A fitting zone and a buffer zone. Only the forces on atoms within the fitting zone are fitted, while the buffer zone serves to shield the fitting zone from MM particles. The use of a relatively large two-zone QM region also reduces the need for diffuse functions in the basis set, as the basis functions of the buffer zone atoms contribute to the modeling of the electron density of the fitting zone atoms.

For each of the 200 conformations, a molecule is randomly selected and designated as the central molecule of the fitting zone. Of all molecules with any atom within 3 Å of the central molecule, five molecules are selected to be included in the fitting zone. Additionally, any molecule within 3 Å of any atom of a fitting zone molecule is assigned to the buffer zone. The QM region comprises both the fitting and buffer zones, with the remaining molecules constituting the MM region. All QM/MM calculations are performed using the ORCA^57^ software package with tight self-consistent field (SCF) convergence criteria.

In the FM step, the QM forces on the fitting region atoms are used to create the force field models using the CReate Your Own Force Field (CRYOFF) program, version 4.4.0, developed in the Wang group.^58^

The parameters were obtained through a multistep process. To reduce the number of nonlinear parameters, the current implementation of AFM in CRYOFF fits only charge products. In the first step of the fitting, atomic charges are determined from the charge products using the Charge Matrix Decomposition (CMD) method.^30^

After the atomic charges have been determined, the repulsion terms are fitted in the second step. Typically, AFM models employ an exponential repulsion term, given by

2 $$U_{\mathrm{rep}}\left(r_{\mathrm{ij}}\right)=Ae^{-\alpha r_{\mathrm{ij}}}$$

where *r*_*ij*_ is the interatomic distance, and *A* and α are fitting parameters. Of these two parameters, *A* is a linear parameter that is solved using singular value decomposition, whereas α is a nonlinear parameter that is optimized using the Simplex method.

The stability of these parameters depends on the placement of the repulsive terms. The inclusion of repulsive interactions between all pairs of atoms can compromise the numerical stability of the fitting process, as multiple solutions with similar performance emerge. This is because different combinations of repulsive strengths can produce comparable forces, such as stronger repulsion between contacting atoms and weaker repulsion between buried atoms versus weaker repulsion between contacting atoms and stronger repulsion between buried atoms. To mitigate this issue, we implemented a parameter-stability based approach to eliminate redundant repulsive terms and improve the robustness of the fitting process.

Following the determination of charges using the CMD method, we performed 100 trial fits with repulsions between all pairs of atoms, subject to an α constraint ranging from 2.0 Å^–1^ to 6.0 Å^–1^. The initial guesses for α were randomly drawn from a uniform distribution within this range. After completing the fittings, we computed the standard deviation of the optimized α values. If the standard deviation for a particular α value exceeded 0.6 Å^–1^, we removed the repulsion term with the largest standard deviation in α. We then conducted another 100 trial fits using the remaining repulsive terms and new initial guesses for α. This iterative process was repeated until all α parameters had a standard deviation of 0.6 Å^–1^ or less.

After the repulsions have been determined, the intramolecular force-field parameters are optimized in a subsequent step. In this step, all intermolecular nonbonded terms are fixed to their previously optimized values. Intramolecular nonbonded interactions are restricted to atoms beyond 1–3 connectivity, and the following terms are fitted: harmonic bond, harmonic angle, and cosine dihedral terms.

After the FM step, the AFM process restarts from the sampling step, using the force field fitted in the previous FM step to perform MD simulations in the sampling step and to model the MM region in the QM/MM step. Each new generation of AFM fits the QM reference forces of up to two previous generations to improve parameter stability. The density and enthalpy of vaporization are computed at each generation to monitor force field convergence. In all cases, convergence is reached at generation 3. The densities and enthalpy of vaporization of each generation of AFM is reported in the Supporting Information. A total of 6 generations are performed for each target molecule, with the final 4 generations being combined in a global fit. The parameter-stability study is only performed twice: once in the first generation and again in the global fit.

As AFM uses simple molecular mechanics energy expressions, similar to traditional force fields, the extra cost of AFM modeling is only incurred during the model development stage. The cost of development is predominantly determined by the cost of the QM/MM calculations. For the development of each model, 1,200 QM/MM calculations are performed over six iterations. Each QM/MM calculation took between 30 min for DMA and 2 h for THF, when running on 20 AMD EPYC cores. More detailed information regarding the cost of AFM model development is provided in the Supporting Information.

The final force field parameters of the three models are provided in the Supporting Information (SI).

## 3 Property Calculations

In order to validate the performance of the AFM models based on B3LYP-D3­(BJ), we compute the following ensemble properties of these molecules and compare them with those obtained from other established force fields, including OPLS-AA (CM1A), the General AMBER Force Field 2 (GAFF2), and GROMOS 54A7.

OPLS-AA­(CM1A) is an improved version of the OPLS-AA model, in which its partial charges are replaced by Continuum Solvation Model 1 charges scaled by 1.14. This combination has been specifically validated to improve the prediction of small molecule hydration free energies while maintaining good reproduction of density and heat of vaporization.

GAFF2 is an updated version of the GAFF force field that incorporates advanced parametrization techniques in its development. It provides comprehensive coverage of chemical systems, with a special focus on noncovalent interactions.

The GROMOS 54A7 force field is an updated iteration of the 54 series GROMOS model, featuring revised parameters aimed at enhancing the accuracy of noncovalent interactions, including hydrogen bonding and van der Waals interactions. However, our results unexpectedly revealed that the all-atom version of the GROMOS 54A7 model performed poorly. Consequently, we opted to utilize the united-atom version instead. The united-atom version demonstrated better agreement with experimental data for all investigated properties when compared to its all-atom counterpart.

The density (ρ), heat of vaporization (Δ*H*_vap_), viscosity (η), self-diffusion constant (*D*) and dielectric constant (ε_0_), free energy of vaporization (Δ*G*_vap_), and boiling temperature (*T*_b_) are computed for each model. The ρ and Δ*H*_vap_ are fundamental properties that are generally fitted during the development of every traditional force field, *D*, ε_0_, Δ*G*_vap_, and *T*_b_ are important parameters that determine the suitability of a solvent for battery applications. Specifically, η, *D* are transport properties related to ion mobility, ε_*s*_ describes how the solvent screens the electric field of ions and directly affects the solvation free energy; Δ*G*_vap_, is related to the equilibrium vapor pressure, together with *T*_*b*_, determines the window of stability of the solvent.

It is worth noting that the AFM models include a −*C*_8_/*r*_8_ dispersion term. However, the long-range correction to energy and stress as implemented in GROMACS does not consider this −*C*_8_/*r*_8_ contribution. A procedure for manually correcting the energy and pressure has been discussed by Nikitin and Wang.^30^ The long-range contribution to pressure due to truncating the *C*_8_ term can be calculated as

3 $$P_{C_{8}}=-\sum \frac{32\pi }{15}\frac{N\times C_{8,\mathrm{ij}}}{V^{2}r_{\mathrm{vdW}}^{5}}$$

where *N* is the number of pairs containing the *C*_8_ dispersion, *r*_vdw_ is the van der Waals cutoff, *V* is the box volume, and *C*_8,*ij*_ refers to the *C*_8_ dispersion parameter between atoms *i* and *j*. The actual pressure, *P*_MD_, is then given by

4 $$P_{\mathrm{MD}}=P_{\mathrm{set}}+P_{C_{8}}$$

where *P*_set_ is the pressure maintained by the GROMACS barostat, which does not account for *P*_*C*_8__. During the simulation *P*_set_ should be adjusted to make *P*_MD_ the target pressure.

Unless otherwise stated, all property measurements are performed using GROMACS version 2019.2 ^59^ with the leapfrog integrator and a time step of 0.5 fs. For NVT simulations, the Nose-Hoover thermostat is used with a relaxation constant of 2 ps. The Parrinello–Rahman barostat with a relaxation time of 5 ns is employed for pressure control in NPT simulations. All simulations are performed in cubic boxes containing 500 molecules.

Due to the lower boiling temperature of DMA, all properties of DMA are determined at 273.15 K unless otherwise specified, while all properties of DMC and THF are computed at 298.15 K. The density ρ is calculated through a 2 ns MD simulation at 1 bar *P*_MD_.

The heat of vaporization is calculated according to

5 $$\Delta H_{\mathrm{vap}}=\left\langle E_{\mathrm{gas}}\right\rangle -\left\langle E_{\mathrm{Liq}}\right\rangle +\mathrm{RT}$$

where ⟨*E*_gas_⟩ is the potential energy of a single molecule in the gas phase, ⟨*E*_Liq_⟩ is the average potential energy per molecule in the liquid phase, *R* is the ideal gas constant, and *T* is the temperature. The ⟨*E*_Liq_⟩ is measured over a 2 ns MD simulation under NPT conditions. The ⟨*E*_gas_⟩ is computed with a 100 ns MD simulation in the same volume as the corresponding liquid box using the Langevin thermostat.

The self-diffusion coefficient *D* was calculated according to the equation

6 $$D=\frac{1}{6}\lim_{t\to \infty }\frac{d}{dt}\left\langle {\left|r_{i}\left(t\right)-r_{i}\left(0\right)\right|}^{2}\right\rangle +\frac{k_{B}T\xi }{6\pi \eta L}$$

where the first term is the Einstein relation, with *r_i_* (*t*) denoting the position of molecules *i* at time *t*. The second term is a finite size correction, as proposed by Yeh and Hummer,^60^ where the geometric constant, ξ, is 2.837297 for a cubic box with length *L*, *k*_B_ is the Boltzmann constant, *T* is the temperature, and η is the shear viscosity of the liquid.

The shear viscosity is related to the fluctuation of the off-diagonal elements of the pressure tensor

7 $$\eta =\frac{1}{2}\frac{V}{k_{B}T}\lim_{t\to \infty }{\left\langle {\left(\int_{t_{0}}^{t_{0}+t}P_{\alpha \beta }\left(t^{\prime }\right){dt}^{\prime }\right)}^{2}\right\rangle }_{t_{0}}$$

where *V* is system volume, *k*_B_ is Boltzmann constant, *T* is temperature, and *P*_αβ_ represents one of the off-diagonal components of the pressure tensor. Because viscosity converges slowly, at least five independent simulations, each with a duration of no less than 20 ns, were performed to improve statistical reliability. To cross-check the reliability of our procedure, the viscosity of DMA was also computed by a double-exponential fit of the Green–Kubo transformation of the off-diagonal elements of the stress tensor according to the procedure of Zhang et al.^61^ The two procedures agree within 0.03 mPa·s, and the cross-check performed in the case of DMA is reported in the SI.

The static dielectric constants (ε_s_) reflects the response of the medium to an external electric field. It consists of two contributions: A high-frequency component (ε_e1_), arising from electron polarization, and a low-frequency component (ε_MD_), resulting from the orientational alignment of molecules in an external field. The ε_s_ is calculated as the product of ε_e1_ and ε_MD_ ^62^

8 $$\varepsilon_{s}=\varepsilon_{\mathrm{el}}\cdot \varepsilon_{\mathrm{MD}}$$

9 $$\varepsilon_{\mathrm{el}}=n_{D}^{2}$$

In our work, the refractive index (*n*_D_) taken to be 1.35 for DMA,^63^ 1.37 for DMC,^64^ and 1.40 for THF.^65^ The ε_MD_ can be computed with GROMACS with the vacuum permittivity, ε_0_, set to one.

10 $$\varepsilon_{\mathrm{MD}}=\varepsilon_{0}+\frac{4\pi \left\langle M^{2}\right\rangle }{3Vk_{B}T}$$

where *V* is the volume of the system, *M* is the dipole moment of the box, which is measured after ensuring that no molecules are divided by the box boundary, and the angle bracket denotes an ensemble average.

The free energy of vaporization (Δ*G*_vap_) of a target molecule is calculated using the alchemical free energy difference between creating the molecule in the gas phase and in the liquid phase. This alchemical free energy is computed using the Bennett Acceptance Ratio (BAR) method,^66^ as implemented in GROMACS. To perform this calculation, the electrostatic interactions are first removed using 20 λ windows, followed by the removal of the short-range nonbonded interactions in 20 windows. A soft-core potential with an α parameter of 0.265 and σ of 1.0 nm is employed to avoid singularities during the decoupling of the short-range nonbonded interactions. Convergence of the calculation is assessed by verifying that the relative entropy between neighboring windows is below 0.2, ensuring that the free energy profiles are smooth and continuous.

The use of pair-specific tabulated potentials in GROMACS requires explicit decoupling of intramolecular nonbonded interactions, which is achieved by setting “couple_intramol” to “yes”. For the gas phase simulations, each λ window was performed using the average box size of the liquid phase, with a single molecule present. Each window was simulated for 10.1 ns under NVT conditions, with the Langevin thermostat^67,68^ used for temperature control. Statistics were collected over the last 10 ns of the simulation. Each liquid phase window was simulated for a total of 2.1 ns, with the final 2 ns used for data collection. This allows for sufficient equilibration and sampling of the relevant configurations.

The knowledge of Δ*G*_vap_ and liquid density at a series of temperatures enables the determination of the boiling temperature (*T*_B_) of the liquid. As

11 $$\Delta G_{\mathrm{vap}}=-\;\mathrm{RT}\ln\left(\frac{\rho_{g}}{\rho_{l}}\right)$$

assuming the vapor phase satisfies the ideal gas law, the vapor pressure (*P*_g_) can be estimated with

12 $$\ln\left(\frac{P_{g}}{P^{{}^{\circ}}}\right)=\ln\left(\frac{\mathrm{RT}\rho_{l}}{P^{{}^{\circ}}}\right)-\frac{{\Delta G}_{\mathrm{vap}}}{\mathrm{RT}}$$

where *P*° is a reference pressure, chosen to be 1 atm. The *T*_B_ can then be determined by correlating ln­(*P*_g_/*P*°) with 1/*T* according to the Gibbs–Helmholtz relation.

In practice, Δ*G*_vap_ is computed at three different temperatures that span a range of 30 K around the experimental *T*_B._ For all three AFM models, the *T*_B_ of the model falls within this 30 K window. When this is not the case, ideally it would be best to create a new 30 K window around the model *T*_B_. However, the additional Δ*G*_vap_ calculations are not performed even if the model *T*_B_ is not within the initial window, since our goal is only to rank the quality of the models. If the *T*_B_ falls out of the window, we already know that the quality of the model is relatively poor at reproducing this property.

## 4 Results and Discussion

The properties of DMA, DMC, and THF predicted by the AFM models trained against B3LYP­(D3-BJ) are summarized in Tables 1 through 3. The experimental reference values, along with predictions from traditional force fields: OPLS-AA (CM1A), GAFF2, and GROMOS 54A7, are also reported.

**Table 1:** Density (ρ) and Heat of Vaporization (Δ*H*_vap_) for the Liquids Studied^a^

	DMA		DMC		THF	
	ρ	Δ*H*_vap_	ρ	Δ*H*_vap_	ρ	Δ*H*_vap_
	kg m^–3^	kJ mol^–1^	kg m^–3^	kJ mol^–1^	kg m^–3^	kJ mol^–1^
EXP.	680 ^63^	26.5 ^69^	1060 ^70^	37.75 ^71^	880 ^72^	∼33 ^63^
OPLS-AA (CM1A)	742 ± 0.6	39.41 ± 0.09	1078 ± 0.3	51.61 ± 0.04	**859** ± 0.3	**32.93** ± 0.04
GAFF2	726 ± 0.5	30.28 ± 0.04	**1047** ± 0.2	44.72 ± 0.04	843 ± 1.7	34.95 ± 0.07
GROMOS	648 ± 1.4	23.81 ± 0.05	1110 ± 0.2	53.39 ± 0.04	762 ± 0.5	27.8 ± 0.03
AFM	**698** ± 0.5	**29.17** ± 0.05	1018 ± 0.9	**36.49** ± 0.02	928 ± 0.5	34.79 ± 0.02

a The values with the best agreement are in bold.

Table 1 summarizes the density (ρ) and heat of vaporization (Δ*H*_vap_) for the three molecules studied. These properties are typically fitted during traditional force field development, and the performance of different models can be evaluated based on their ability to reproduce these properties.

The B3LYP-D3­(BJ)-based AFM models performed exceptionally well, providing the best agreement with experimental values for three out of six properties. For DMA, the AFM model showed excellent agreement with both ρ and Δ*H*_vap_. In contrast, the GAFF2 model excelled in predicting ρ for DMC, but its error in Δ*H*_vap_ was quite substantial (7 kJ/mol). Similarly, OPLS-AA­(CM1A) accurately predicted ρ, but significantly overestimated Δ*H*_vap_. The AFM model, however, provided a very good ρ and only a minor deviation in Δ*H*_vap_, making it the best model for DMC in terms of these properties.

For THF, both OPLS-AA­(CM1A) and GAFF2 models outperformed the B3LYP­(D3-BJ)-based AFM model in predicting ρ and Δ*H*_vap_. This is not surprising, given that THF or similar molecules may have been fit directly during the development of these models. Although the AFM model ranked third in terms of performance, its error in ρ was relatively small (5%), and the 2 kJ/mol deviation in Δ*H*_vap_ can be considered excellent agreement.

It is worth noting that the AFM model only fits DFT references, making it challenging to distinguish between the contributions of the imperfect fitting process and the intrinsic limitations of the DFT functional to the observed deviations. Nevertheless, the AFM model’s performance across the three molecules demonstrates its reliability for predicting basic thermodynamic properties and the accuracy of the selected reference functional.

Table 2 summarizes two transport properties, viscosity (η) and diffusion constant (*D*), for the molecules studied. For DMA, no experimental values for η and *D* are available. However, the diffusion constants of monomethylamine (MMA) and trimethylamine (TMA) have been measured at 273 K and various pressures.^71,73^ By extrapolating these measurements to 1 bar, we obtain estimated diffusion constants of 5.18 × 10^–9^ m^2^s^–1^ for MMA and 4.99 × 10^–9^ m^2^s^–1^ for TMA. Assuming that the *D* of DMA falls within this range, the AFM model provides the best agreement, underestimating the true value by 0.2 to 0.4 × 10^–9^ m^2^s^–1^.

**Table 2:** Viscosity (η), Diffusion Constant (*D*) for the Liquids Studied^a^

	DMA		DMC		THF	
	η	*D*	η	*D*	η	*D*
	mPa s	10^–9^ m^2^ s^–1^	mPa s	10^–9^ m^2^ s^–1^	mPa s	10^–9^ m^2^ s^–1^
EXP.	N.A^71^	4.99–5.18 ^73^	0.55 ^70^	2.6 ^74^	0.46 ^72^	3.0 ^74^
OPLS-AA (CM1A)	0.99 ± 0.26	1.08 ± 0.01	1.37 ± 0.30	0.75 ± 0.03	0.55 ± 0.06	2.69 ± 0.09
GAFF2	0.38 ± 0.01	3.87 ± 0.10	0.91 ± 0.13	1.34 ± 0.02	0.53 ± 0.04	**2.81** ± 0.14
GROMOS	0.20 ± 0.03	6.79 ± 0.20	1.20 ± 0.36	0.92 ± 0.04	0.27 ± 0.07	5.04 ± 0.23
AFM	0.23 ± 0.02	**4.78** ± 0.06	**0.59 ± 0.06**	**2.31** ± 0.05	**0.45 ± 0.06**	2.48 ± 0.07

a The values with the best agreement are in bold.

For DMC, the AFM model also yields the best agreement for both η and *D*, underestimating the diffusion constant by 0.3 × 10^–9^ m^2^s^–1^. For THF, while the AFM model provides good agreement for η, it underestimates *D*, by 0.5 × 10^–9^ m^2^s^–1^, which is slightly larger than those observed for DMA and DMC. Both OPLS-AA (CM1A) and GAFF2 models give better agreement for the *D* of THF.

It is important to consider that the diffusion constants were obtained from classical simulations, which neglect quantum nuclear effects. Quantum tunneling is expected to enhance diffusion, and the agreement between the AFM-predicted diffusion constant and the true value is anticipated to improve once quantum nuclear effects are taken into account.

Table 3 presents the static dielectric constant (ε_*s*_), boiling temperature (*T*_B_), and free energy of vaporization (Δ*G*_vap_) for the three molecules in their liquid state. These properties are closely related to the performance of these compounds as battery solvents, with Δ*G*_vap_ being directly related to vapor pressure. A solvent with higher Δ*G*_vap_ and *T*_B_ would be more suitable for operation at elevated temperatures.

**Table 3:** Static Dielectric Constant (ε_s_), Boiling Temperature (*T*_b_) and Free Energy of Vaporization (Δ*G*_vap_) for the Liquids Studied^a^

	DMA			DMC			THF		
	ε_s_	*T*_b_	Δ*G*_vap_	ε_s_	*T*_b_	Δ*G*_vap_	ε_s_	*T*_b_	Δ*G*_vap_
		K	kJ/mol		K	kJ/mol		K	kJ/mol
EXP.	6.3	280	13.61	3.2 ^75^	363	20.94	7.6 ^72^	339	17.89
OPLS-AA (CM1A)	19.5 ± 0.18	312	17.55 ± 0.48	**2.7 ± 0.01**	428	28.68 ± 0.09	11.4 ± 0.06	334	**16.75** ± 0.47
GAFF2	12.8 ± 0.00	286	14.63 ± 0.04	**2.7 ± 0.00**	**375**	**23.20** ± 0.32	**9.9 ± 0.02**	349	20.22 ± 0.19
GROMOS	**6.6 ± 0.01**	277	13.12 ± 0.14	4.0 ± 0.02	444	31.30 ± 0.46	10.0 ± 0.02	324	16.05 ± 0.33
AFM	12.7 ± 0.12	**279**	**13.59** ± 0.28	2.6 ± 0.02	346	18.07 ± 0.09	10.8 ± 0.05	**343**	19.11 ± 0.19

a The values with the best agreement are in bold.

In comparison to empirical force fields, the AFM model excels in predicting *T*_B_ and Δ*G*_vap_ for DMA, providing the best agreement with experimental values. For THF, the AFM model predicts *T*_B_ with high accuracy, overestimating it by only 4 K, which is the best agreement among the four models. The AFM model overestimates Δ*G*_vap_ by 1.2 kJ/mol, which should be considered excellent agreement. This overestimation is consistent with its slight overestimation of ρ and Δ*H*_vap_. This suggests that the AFM model may be slightly overestimating the energy of cohesion in the liquid state.

In comparison, OPLS-AA (CM1A) underestimates Δ*G*_vap_ for THF by only 1.1 kJ/mol, providing the best agreement for this property. This is consistent with OPLS-AA (CM1A) giving the best agreement for ρ and Δ*H*_vap_ for THF.

For DMC, GAFF2 achieved the best agreement, slightly overestimating both *T*_B_ and Δ*G*_vap_. The AFM model shows similar performance to GAFF2 but underestimates both properties. This is consistent with the AFM model’s slight underestimation of ρ and Δ*H*_vap_. Interestingly, GAFF2 substantially overestimates Δ*H*_vap_ while giving a slightly lower ρ. We hypothesize that GAFF2 overestimates intermolecular attraction between DMC molecules but underestimates their size, leading to a cancellation of errors that fortuitously results in a smaller error in *T*_B_ and Δ*G*_vap_.

The AFM models encounter their greatest challenge in accurately reproducing the static dielectric constant (ε_s_). Specifically, they overestimate ε_s_ by a factor of 2 for DMA and by 50% for THF, while underestimating it by 20% for DMC. In comparison to empirical force fields, such as GAFF2 and GROMOS, across all three molecules, the AFM model’s performance is slightly inferior to the best empirical models. Only OPLS-AA (CM1A) exhibits worse agreement than the AFM models.

As AFM provides excellent predictions of other properties, we argue that the failure to accurately predict *ε*_s_ does not reflect an inability of the AFM models to capture the underlying potential energy surface. Instead, the pairwise additive potentials created have intrinsic limitations in modeling dielectric response.

Our approach to capturing the high-frequency ε_e1_ as a multiplicative factor relies on the assumption that there is no correlation between orientational polarization and electronic polarization. However, this approximation may not hold, potentially requiring models that explicitly account for polarizability to accurately determine *ε*_s_.

Although the AFM model does not excel in reproducing ε_s_, its performance is still comparable to that of GAFF2 and GROMOS. Therefore, it is essential to consider the AFM model’s overall performance in the context of other properties, where it has demonstrated excellent agreement with experimental values.

## 5 Summary and Conclusion

While traditional force field development focuses on reproducing experimental properties, AFM has a distinct objective: To accurately reproduce the condensed-phase forces of a reference electronic structure method. In essence, AFM models aim to map an electronic structure potential energy surface (PES) onto a molecular mechanics-based energy expression. This mapping enables simulations to be performed on an approximated electronic structure PES at a significantly lower computational cost. The reduced computational cost afforded by AFM allows electronic structure methods to be applied to larger systems, enabling the prediction of finite-temperature properties that require extensive sampling of the conformational space. For instance, AFM models can predict the *T*_B_, which is not directly accessible through DFT reference calculations. By providing a more efficient and accurate means of modeling complex systems, AFM enables researchers to explore a wider range of phenomena and properties that are crucial in understanding material behavior.

To evaluate the performance of B3LYP-D3­(BJ)-based AFM models, we calculated a range of properties and compared them to those predicted by three recent, high-quality force fields: OPLS-AA (CM1A), GAFF2, and the 54A7 parametrization of GROMOS. If a force field accurately captures intermolecular interactions and provides a reliable partition function, it should be able to predict all properties of the molecule simultaneously with high accuracy.

Table 4 shows the absolute percentage error (APE), which is defined as

13 $$\mathrm{APE}=\frac{1}{M}\sum_{i=1}^{M}\left|\frac{X_{i,\mathrm{calc}}-X_{i,\exp}}{X_{i,\exp}}\right|\times 100\%$$

where the sum is over the *M* types of molecules studied. We excluded DMA when evaluating the percentage error of η and *D*, as the corresponding experimental values are unknown or estimated.

**Table 4:** Average Error (%) with Respect to Experimental Results for Studied Molecules

	ρ	Δ*H*_vap_	η	*D*	***ε***_**0**_	*T*_b_	*G*_vap_	Ave.
OPLS-AA(CM1A)	4.3	28.4	84.3	40.70	91.7	10.3	24.1	40.5
GAFF2	4.3	12.8	40.3	27.4	49.7	2.9	10.4	21.1
GROMOS	7.6	22.5	79.7	66.3	20.5	9.3	21.1	32.4
AFM	4.1	6.2	4.7	14.2	54.2	2.0	6.9	13.2

The mean of the absolute percentage errors (MAPE) for each property is reported in the last column as a measure of the model’s quality. While it is challenging to produce a quantitative comparison between different models, MAPE provides a measure of performance that emphasizes model consistency. A model that produce small errors across all properties would give a smaller MAPE when compares to a model that give excellent agreement only for a few properties.

Among the empirical models, GAFF2 showed the lowest mean absolute percentage error of 21%. In contrast, the AFM models fitted against B3LYP-D3­(BJ) achieved a significantly lower average error of 13%, outperforming the best empirical model by a considerable margin. Notably, the AFM model was developed without fitting any experimental properties, demonstrating the accuracy of the underlying B3LYP-D3­(BJ) PES for such battery solvents and the robustness of the fitting procedure.

The AFM models demonstrate the most significant improvement over empirical models in predicting transport properties, specifically viscosity (η) and diffusion constant (*D*). However, the AFM models perform relatively poorly compared to GROMOS or GAFF2 in reproducing the static dielectric constant (ε_*s*_).

This poor performance suggests that accurately capturing the dielectric response of a solvent molecule may be challenging without explicit modeling of polarization effects. The computation of *ε*_*s*_ involves applying a multiplicative factor that accounts for the electronic contribution to the dielectric constant, as argued by Leontyev and Stuchebrukhov.^62^ Nevertheless, this approach may be insufficient, if electronic polarization is strongly coupled with molecular rotation. Using a fixed charge model to compute the rotational dielectric constant can lead to deficiencies. To improve the prediction of dielectric response, future work will investigate fitting a polarizable potential with AFM, which may help to improve the prediction of dielectric response.

In conclusion, this study has successfully demonstrated the potential of using the AFM method to perform DFT-based simulations for various battery solvents. The resulting models exhibit exceptional accuracy for most properties, underscoring the reliability of the B3LYP-D3­(BJ) method and the effectiveness of AFM fitting.

Although the use of fixed charge models in this study may have limited the ability to capture dielectric response, this investigation establishes AFM as a powerful tool for predicting physical properties of popular solvents in battery design. This work lays the groundwork for predicting properties of novel solvents or solvent mixtures, which is essential for advancing research in fields such as chemistry and materials science.

The findings of this study have significant implications for these fields, where accurate predictions of material behavior are crucial for understanding complex phenomena and designing new materials and systems. By providing a reliable means of simulating solvent properties, AFM can facilitate the development of more efficient and safe energy storage solutions, ultimately contributing to the advancement of battery technology.

## Supplementary Material



## Data Availability

The data underlying this study are available in the published article and its Supporting Information. The GROMACS input files for the three models developed are available at https://wanglab.uark.edu/Models.htm.

## Notes

Additional details of the convergence analyses, computational cost of AFM, force-field parameters, and validation of the viscosity calculations (PDF)
