# Supplementary material for: Predicting Properties of Key Ingredients of Battery Solvents with Density Functional Theory and Adaptive Force Matching
Source: J Chem Theory Comput. 2026 Aug 4;22(16):8521–30. doi: 10.1021/acs.jctc.6c00942 (PMC13523628; doi:10.1021/acs.jctc.6c00942)
Supplement: Supplementary file 1 [file ct6c00942_si_001.pdf]

Supporting Information

Predicting Properties of Key Ingredients of Battery Solvents with Density Functional Theory and Adaptive Force Matching

Yang Wei; Raymond Weldon and Feng Wang*

Department of Chemistry and Biochemistry,
University of Arkansas, Fayetteville, Arkansas 72701, USA

Convergence of AFM Generations

Table S1. The densities and enthalpy of vaporization of each generation of AFM

Iteration	DMA		DMC		THF	
	ρ kg m^{-3}	ΔH_{vap} kJ mol^{-1}	ρ kg m^{-3}	ΔH_{vap} kJ mol^{-1}	ρ kg m^{-3}	ΔH_{vap} kJ mol^{-1}
01	739	38.71	1084	50.6	866	33.14
02	681	29.92	1030	35.72	911	33.66
03	662	26.81	1013	34.94	922	34.54
04	673	26.57	1022	35.14	924	34.58
05	687	28.51	1022	35.45	917	34.22
06	687	28.44	1029	35.56	924	34.97

Gen 1 was sampled with the initial guess force field, and we included the last two generations of QM/MM calculations in each generation of AFM. Therefore, the first generation that includes the full number of QM/MM reference forces is Generation 3. All generations from 3 to 6 led to consistent densities and enthalpies of vaporization and are considered converged.

Computational Cost of AFM

For each generation, 200 frames were extracted from MD simulations performed at two different temperatures. For each frame, a single-point QM/MM calculation was carried out using OCRA with density fitting DFT and B3LYP-D3(BJ)/def2-TZVP. Six generations of AFM were performed for each model, which required 1,200 QM/MM calculations. The average time for each calculation is summarized in Table S2.

Table S2. AFM performance for the studied molecules

	Number of Cores	Ave. QM Atoms	Ave. Wall Time (mins)
DMA	20	1035	37
DMC	20	1039	86
THF	20	1131	99

Force field parameters

$$U_{ij} = \sum_{ij}\left(\frac{q_i q_j}{4\pi\epsilon_0 r_{ij}} + Ae^{(-\alpha\times r)} + \frac{C_6}{r_0^6+r_{ij}^6} + \frac{C_8}{r_0^8+r_{ij}^8}\right) + \sum_i \frac{k_b}{2}(r - r_e)^2 + \sum_i \frac{k_\theta}{2}(\theta - \theta_e)^2 + \sum_i V_D(1 + \cos(m \times \omega - \delta))$$

Table S3. Atomic charges for DMA obtained with the charge matrix decomposition (CMD) method

	**CMD**
C1	0.1898
H1	-0.0026
N1	-0.7399
H2	0.3759

Table S4. Repulsion Parameters of DMA

Pair	A<br>kcal/mol	α<br>$Å^{-1}$
H2 H2	38424.66	5.91
H2 H1	776.61	3.84
H2 C1	1404.56	2.96
H1 H1	834.54	3.57
H1 C1	46605.54	4.46
H1 N1	51991.79	4.82
C1 C1	33645.53	2.83
C1 N1	443093.87	4.01
N1 N1	1247282.40	4.43

Table S5. Dispersion Parameters of DMA

Pair	C6<br>Kcal mol^{-1} Å^{-6}	C8<br>Kcal mol^{-1} Å^{-8}	r_0<br>Å^{-1}
C1 C1	-1447.73	-36007.64	2.044
C1 N1	-611.32	-7311.58	2.032
N1 N1	-576.27		2.019

Table S6. Bonds parameters of DMA

Bonds	r_e<br>(nm)	k_b<br>(kj mol^{-1} nm^{-2})
C1-H1	0.10967	291206.87
C1-N1	0.14566	230545.70
N1-H2	0.10128	404507.48

Table S7. Angles parameters of DMA

Angles	θ_e<br>(°)	k_θ<br>(kj mol^{-1} rad^{-2})
C1-N1-C1	111.57939	541.1095
H1-C1-N1	109.90872	427.2296
H2-N1-C1	108.26241	410.5152
H1-C1-H1	106.71144	295.8850

Table S8. Dihedral parameters of DMA

Dihedral	δ_e<br>(°)	V_D<br>(kj mol^{-1})	m
H1-C1-N1-H2	0.00	0.55800	3

Table S9. Atomic charge for THF from charge matrix decomposition

	CMD
O1	-0.3500
C1	0.0941
C2	0.0185
H1	0.0201
H2	0.0111

Table S10. repulsion parameters of THF

Pair	A<br>kcal/mol	α<br>$Å^{-1}$
H1-H1	620.99	3.37
H1-H2	621.12	3.39
H1-C1	16470.52	4.17
H1-C2	81082.46	4.80
H1-O1	69931.44	5.33
H2-H2	677.37	3.43
H2-C1	26876.70	4.38
H2-C2	46091.79	4.64
H2-O1	54345.97	5.32
C1-C1	37180.90	2.92
C1-C2	31543.45	2.82
C1-O1	171745.58	3.89
C2-C2	56240.58	3.00
C2-O1	320165.74	4.12

Table S11. Dispersion parameters of THF

Pair	C6<br>kcal mol^{-1} $Å^{-6}$	C8<br>kcal mol^{-1} $Å^{-8}$	r_0<br>$Å^{-1}$
C1-C1	-1045.50	-33203.86	2.044
C1-C2	-777.05	-38977.42	2.044
C2-C2	-542.40	-37234.42	2.044
O1-C1	-30.25	-5028.03	1.997
O1-C2	-60.41	-890.11	1.997

Table S12. Bonds parameters of THF

Bonds	r_e (nm)	k_b (kj mol^{-1} nm^{-2})
C1-H1	0.11	290303.44
C2-H2	0.11	301279.91
O1-C1	0.14	218985.36
C1-C2	0.15	185484.02
C2-C2	0.15	188005.69

Table S13. Angles parameters of THF

Bonds	r_e (nm)	k_b (kj mol^{-1} nm^{-2})
C1-O1-C1	111.2325	565.4808
O1-C1-C2	108.2555	834.9505
C1-C2-C2	105.0859	686.5774
H1-C1-H1	105.1833	292.8565
H2-C2-H2	98.7320	298.7974
H1-C1-C2	109.0072	372.6279
H2-C2-C1	104.3772	399.6414
H2-C2-C2	104.5951	389.7293
H1-C1-O1	106.9997	456.5438

Table S14. Dihedral parameters of THF

Dihedral	δ_e (°)	V_D (kj mol^{-1})	m
O1-C1-C2-C2	0.0000	5.2625	3
O1-C1-C2-C2	0.0000	5.2625	3
C1-C2-C2-C1	0.0000	-3.7868	3
C2-C1-O1-C1	0.0000	3.1330	3
C2-C1-O1-C1	0.0000	3.1330	3

Table S15. Atomic charge for DMC from charge matrix decomposition

	**CMD**
C1	0.7291
O1	-0.5551
O2	-0.3288
C2	-0.1287
H1	0.1235

Table S16. repulsion parameters of DMC

Pair	A<br>kcal/mol	α<br>$Å^{-1}$
H1-H1	1374.79	4.05
H1-C1	4057.60	3.61
H1-C2	5191.97	3.50
H1-O1	6798.06	4.04
H1-O2	11216.32	4.24
C1-C2	6358.12	2.79
C1-O1	712515.76	5.04
C2-C2	58732.72	3.05
C2-O1	37295.15	3.37
C2-O2	104875.20	3.83
O1-O1	6636796.60	5.60
O1-O2	8496290.70	5.80
O2-O2	1030624.50	5.00

Table S17. Dispersion parameters of DMC

Pair	C6<br>kcal mol^{-1} $Å^{-6}$	C8<br>kcal mol^{-1} $Å^{-8}$	r_0<br>$Å^{-1}$
C1-C2	-1209.97		2.044
C1-O1	-292.69		1.997
C2-C2	-1567.77	-35777.84	2.044
O1-C2	-760.83		1.997
O1-O1	-263.09		1.949
O1-O2	-147.61		1.949
O2-C2	-396.00		1.997
O2-O2	-120.20		1.949

Table S18. Bonds parameters of DMC

Bonds	r_e<br>(nm)	k_b<br>(kj mol^{-1} nm^{-2})
C1-O1	0.12	680384.3
C1-O2	0.13	321737.1

O2-C2	0.14	231681.6
C2-H1	0.11	310307.3

Table S19. Angles parameters of DMC

Bonds	r_e (nm)	k_b (kj mol^{-1} nm^{-2})
O1-C1-O2	149.1689	595.5321
C1-O2-C2	101.3319	500.9815
O2-C1-O2	135.8869	635.0506
O2-C2-H1	104.5506	458.8500
H1-C2-H1	106.1857	305.1273

Table S20. Dihedral parameters of DMC

Dihedral	δ_e (°)	V_D (kj mol^{-1})	m
O1-C1-O2-C2	180.0	14.3069	2
C2-O2-C1-O2	180.0	14.6681	2

Validation of Shear Viscosity Calculation

As a converged value for the shear viscosity η is difficult to obtain, the η of dimethylamine (DMA) was calculated using the Green-Kubo approach and fitted to the double exponential form according to Zhang.[1] Using the AFM model for DMA, η was computed using the Green-Kubo relationship:

$$\eta = \frac{V}{k_{\mathrm{B}}T}\int_{t_0}^{t_0+t} < P_{\alpha\beta}(t')P_{\alpha\beta}(0) > dt' \quad (1)$$

where *V* is the system volume, k_{B} is the Boltzmann constant, *T* is temperature, $P_{\alpha\beta}$ denotes the element αβ of the pressure tensor, and the angle bracket indicates the ensemble average.

A total of 100 independent NVT trajectories were generated at the target temperature, each with a duration of 5 ns, and the final 4 ns were used for analysis. The pressure tensor components were recorded every 2.5 fs. The running viscosity ⟨η(t)⟩ and its standard deviation σ(t) were obtained by averaging over all trajectories. The time-dependent standard deviation was fitted to a power-law function:

$$\sigma(t) = At^{b} \quad (2)$$

where A and b are fitting parameters. As shown in Figure S1A. the corresponding *A* and *b* parameter was found to be 0.005 $\mathrm{cP \cdot ps^{-0.507}}$and 0.507.

The cutoff time t_{cut} was defined as the time at which $\sigma(t)$ reaches 40% of $\langle\eta(t)\rangle$. To reduce the influence of short-time fluctuations, the first 2 ps of data were excluded. The remaining data in the interval $2\,\mathrm{ps} \le t \le t_{\mathrm{cut}}$ were fitted using a weighting function $1/t^{b}$ to a double-exponential model,

$$\eta(t) = A\alpha\tau_1\left(1 - e^{\frac{-t}{\tau_1}}\right) + A(1-\alpha)\tau_2\left(1 - e^{\frac{-t}{\tau_2}}\right) \quad (3)$$

where A, α, τ_1, and $\tau_2$are fitting parameters. Figure S1B shows the shear viscosity of DMA at 273.15 K obtained using this approach with a value of 0.256 mPa·s, which is close to the value of 0.233 mPa·s obtained using Eq. 7 of the main txt.

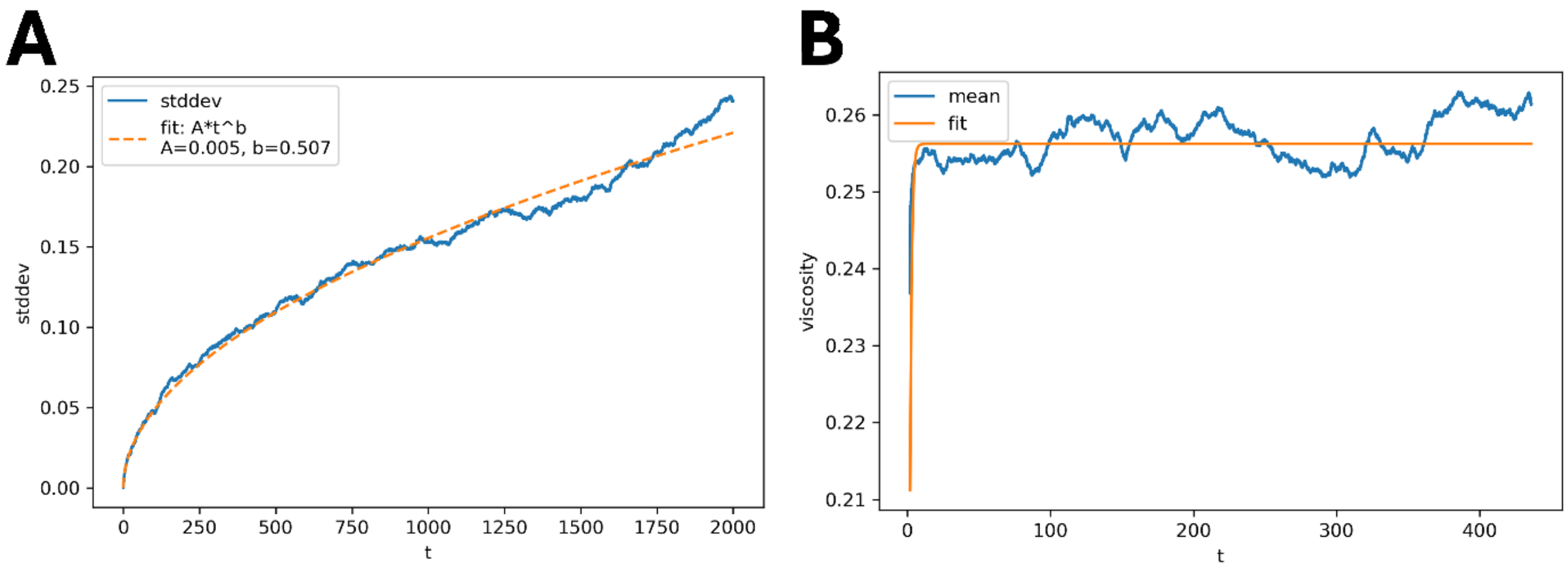

Figure S1. Fitting results for DMA at 273.15 K A). the standard deviation to the power law function B), the averaged running integral to the double exponential function with $1/t^b$ weight

References

(1) Zhang, Y.; Otani, A.; Maginn, E. J. Reliable viscosity calculation from equilibrium molecular dynamics simulations: A time decomposition method. *J. Chem. Theory Comput.* **2015**, *11* (8), 3537-3546.
